# Identification of the novel markers of PPAR signalling affecting immune microenvironment and immunotherapy response of lung adenocarcinoma patients

**DOI:** 10.1111/jcmm.17877

**Published:** 2023-08-09

**Authors:** Wei Zhang, Junhui Liu, Xin Ren, Zhengbin Zhang, Meilan Zhou, Yuehua Li, Jianjie Wang, Quan Li, Qi Zhu, Gang Wu

**Affiliations:** ^1^ Tuberculosis ward No1, Wuhan Pulmonary Hospital Wuhan Institute for Tuberculosis Control, Affiliated to Janghan University Wuhan China; ^2^ Department of Tuberculosis Control, Wuhan Pulmonary Hospital Wuhan Institute for Tuberculosis Control Affiliated to Janghan University Wuhan China; ^3^ Wuhan Pulmonary Hospital Wuhan Institute for Tuberculosis Control Wuhan China; ^4^ Medical department, Wuhan Pulmonary Hospital Wuhan Institute for Tuberculosis Control, Affiliated to Janghan University Wuhan China

**Keywords:** GEO, lung adenocarcinoma, PPAR, prognosis, TCGA

## Abstract

Peroxisome proliferator‐activated receptors (PPARs) are essential for cellular physiological processes. However, there is less research on the PPAR‐related genes in lung adenocarcinoma (LUAD). Open‐access data were get from the cancer genome atlas (TCGA) and gene expression omnibus (GEO) databases. All the analysis were conducted in the R software based on different R packages. In this study, we gauged the PPAR score employing a set of 72 PPAR‐associated genes and probed the biological impact of this score on lung adenocarcinoma (LUAD). Subsequently, we established a unique signature composed of eight PPAR‐related genes (ANGPTL4, ACSL3, ADIPOQ, FABP1, SLC27A1, ACOX2, PPARD and OLR1) to forecast the prognosis of LUAD. The signature's effectiveness in predicting survival was validated through the receiver operating characteristic curve in the TCGA‐LUAD cohort. As per the pathway enrichment analysis, several crucial oncogenic pathways and metabolic processes were enriched in high‐risk individuals. Further, we observed that these high‐risk patients exhibited heightened genomic instability. Additionally, compared to the low‐risk cohort, high‐risk patients demonstrated diminished immune components and function. Intriguingly, high‐risk patients exhibited a potential heightened sensitivity to immunotherapy and certain drugs, including Gefitinib, Afatinib, Erlotinib, IAP_5620, Sapitinib, LCL161, Lapatinib and AZD3759. The prognosis model based on eight PPAR‐related genes has satisfactory prognosis prediction efficiency. Meanwhile, our results can provide direction for future studies in the relevant aspects.

## 1 INTRODUCTION

Lung cancer, one of the most lethal malignancies, continues to hold the highest incidence rate globally.^1^ Alarmingly, only a mere 20% of lung cancer patients manage to survive beyond 5 years.^2^ Lung cancer can be categorized into small cell carcinoma and non‐small cell carcinoma, with their respective five‐year survival rates standing at 19% and 23%.^3^ Approximately 75% of patients were initially diagnosed as having progressed to advanced stages with metastatic disease, largely because current detection tools do not adequately account for tumour heterogeneity, thus affecting diagnostic accuracy.^4^ The strong correlation between lung cancer and familial clustering, as well as specific gene mutations, warrants further investigation, as this understanding could illuminate the mechanisms through which gene mutations induce changes.^5^ The survival rate of lung cancer has seen only a marginal improvement in recent years, owing largely to the advent of molecularly targeted therapies, such as immune checkpoint inhibitors, rather than enhancements in screening methods.^4^ Identifying biomarkers for early diagnosis and therapeutic targeting is as crucial as targeting specific factors such as epidermal growth factor receptors, ROS proto‐oncogene 1 rearrangement and anaplastic lymphoma kinases.

Peroxisome proliferator‐activated receptors (PPARs) are recognized as nuclear receptors capable of regulating the expression of certain target genes. They are available in three distinct forms: PPARα (NR1C1), PPARβ/δ (NR1C2) and PPARγ (NR1C3).^6^ Possessing the ability to heterodimerize with RXR, PPARs exhibit diverse functionalities when they interact with various cofactors and ligands across different tissues.^7^ PPARγ is found in human lung cancer cell lines and its expression levels have been correlated with the differentiation status relevant to carcinogenesis and the OS rate in lung cancer patients.^8^, ^9^ An array of PPARγ ligands can modulate tumour growth and progression by influencing the WNT, MAPK, NF‐kB and TGF‐β signalling pathways in lung cancer cells and stromal cells, in a PPARγ‐dependent fashion. PPAR ligands also have PPARγ‐independent mechanisms that crucially contribute to anti‐cancer activities.^10^ Adenocarcinomas that are well‐differentiated display higher PPARγ expression levels compared to those that are poorly differentiated.^11^ However, in lung cancer cells, due to altered PPARγ functional domains and a shortage of ligands, PPARγ tends to accumulate in the cytoplasm instead of being activated.^12^ The activation of PPAR can be used as an anti‐tumour signal, curbing cancer cell proliferation, metastasis, and growth, and accelerating cellular apoptosis.^8^

Bioinformatics is a powerful tool for screening molecules. Here, we gauged the PPAR score employing a set of 72 PPAR‐associated genes and probed the biological impact of this score on Lung Adenocarcinoma (LUAD). Subsequently, we established a unique signature composed of eight PPAR‐related genes (ANGPTL4, ACSL3, ADIPOQ, FABP1, SLC27A1, ACOX2, PPARD and OLR1) to forecast the prognosis of LUAD. The signature's effectiveness in predicting survival was validated through the receiver operating characteristic curve in the TCGA‐LUAD cohort. As per the pathway enrichment analysis, several crucial oncogenic pathways and metabolic processes were enriched in high‐risk individuals. Further, we observed that these high‐risk patients exhibited heightened genomic instability. Additionally, compared to the low‐risk cohort, high‐risk patients demonstrated diminished immune components and function. Intriguingly, high‐risk patients exhibited a potential heightened sensitivity to immunotherapy and certain drugs, including Gefitinib, Afatinib, Erlotinib, IAP_5620, Sapitinib, LCL161, Lapatinib and AZD3759.

## 2 METHOD

### 2.1 Acquisition of public data

The RNA expression data and clinical characteristics of LUAD patients were collected from the cancer genome atlas (TCGA) and the gene expression omnibus (GEO).^13^, ^14^ The gene expression microarray dataset GSE31210 was selected and downloaded for validation.^15^ Only patients with OS of more than 30 days would be incorporated into further analysis. For TCGA‐LUAD cohorts, we downloaded mRNA expression profiles in Transcripts per million (TPM) values. For the GSE31210 dataset, we transformed gene symbols from probe IDs according to platform annotation files and used normalized expression values to validate the model. Differentially expressed genes (DEGs) analysis was performed using the limma package under the specific threshold value.^16^

### 2.2 Evaluating PPAR score in the TCGA‐LUAD cohort

The list of PPAR‐related genes was collected from previous studies.^17^ Single sample gene set enrichment analysis (ssGSEA) algorithm was used to quantify PPAR score in tumour tissue based on gene expression profiles.^18^

### 2.3 Development of the prognostic gene signature and external validation

We performed a univariate Cox analysis of OS to identify PPAR‐associated genes with prognostic significance, with a *p*‐value cut‐off of <0.05 indicating statistical relevance. To construct a prognostic gene signature and mitigate the likelihood of overfitting, both LASSO and multivariate Cox regression analyses were employed.^19^ Based on the median value of the risk scores, patients were categorized into high‐risk and low‐risk groups. The predictive accuracy of the prognostic gene signature was subsequently validated using the GSE31210 dataset. We also compared the expression levels of each gene between tumour and normal tissues. By integrating the clinical information with the risk scores, a nomogram plot was created.

### 2.4 Biological function analysis

Gene ontology (GO) and Kyoto Encyclopaedia of Genes and Genomes (KEGG) analysis were conducted utilizing the cluster profiler package based on the DEGs between the high‐ and low‐risk groups.^20^ gene set enrichment analyses (GSEA) were conducted to screen enriched terms based on Hallmark gene sets.^21^

### 2.5 Genomic instability analysis

Tumour mutation burden (TMB) and microsatellite instability (MSI) were directly obtained from the TCGA database. Copy number variations (CNVs) represent notable aberrations that modify gene expression, thereby instigating tumorigenesis and disease progression. Employing GISTIC 2.0, we analysed the critical aberrant genomic regions within tumour samples from LUAD patients.

### 2.6 Tumour immune infiltration analysis

Infiltrated immune cells play an important part in tumour immunotherapies. ssGSEA and ESTIMATE algorithms were used to quantify the score of immune cells and immune functions to compare the infiltrated immune cells in tumour samples with different risk score.^18^

### 2.7 Immunotherapy and drug sensitivity

Tumour immune dysfunction and exclusion (TIDE) algorithm is a great method to predict the immunotherapy response based on gene expression levels and a lower TIDE score was correlated with better immunotherapy treatment.^22^ Besides, the SubMap algorithm was used to evaluate the response of LUAD patients to anti‐PD1 and anti‐CTLA4 therapy. The drug sensitivity of LUAD patients was predicted by pharmacogenomic data from the genomics of drug sensitivity in cancer (GDSC) database.^23^

### 2.8 Statistical analysis

All statistical analysis was conducted in the R software and the comparisons with *p* value less than 0.05 were considered as statistically significant.

## 3 RESULTS

**FIGURE 1:**
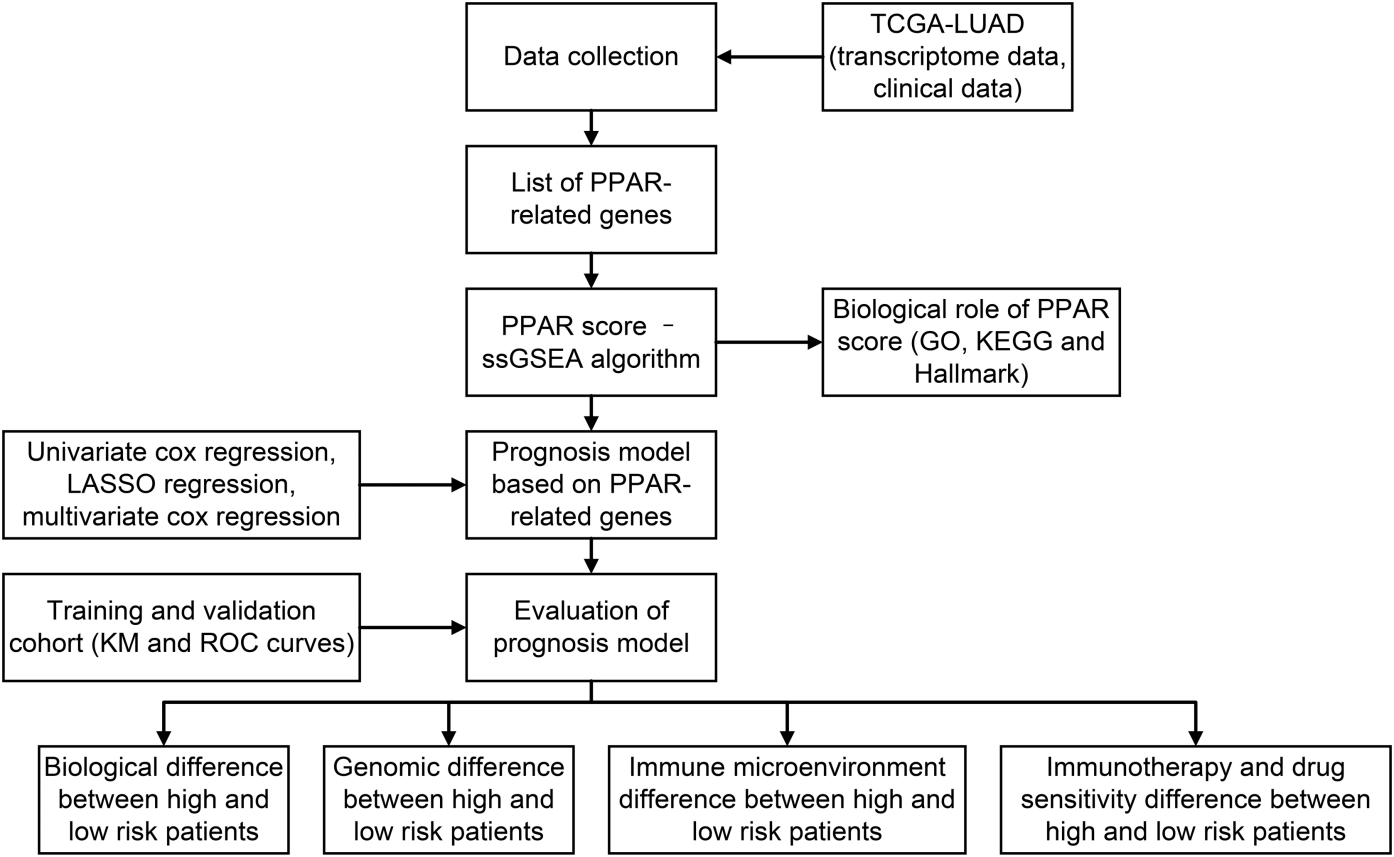
The flow chart of whole study.

Figure 1 illustrated the whole flow chart of this study.

### 3.1 Investigation of PPAR‐related genes

**FIGURE 2:**
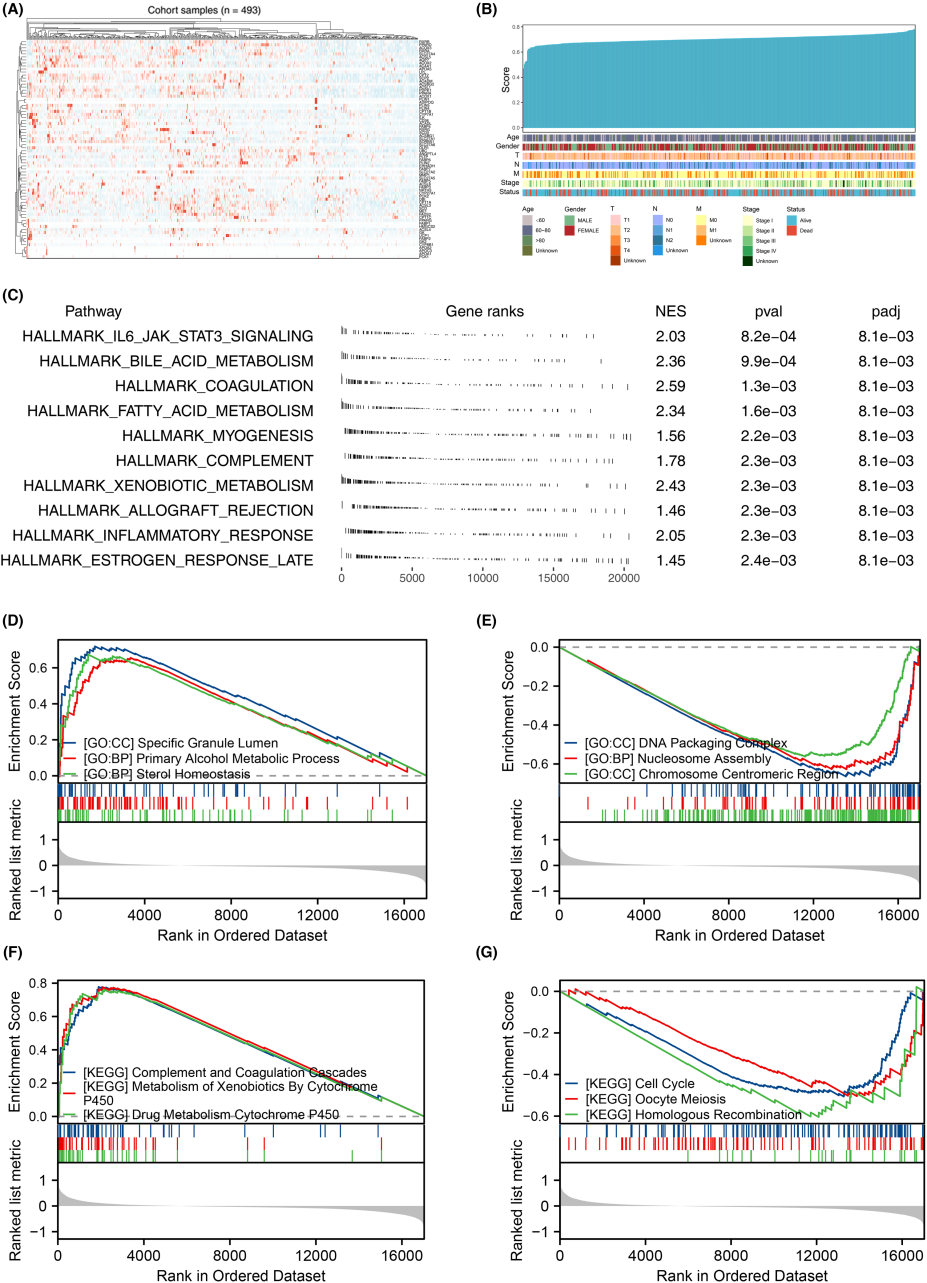
Quantification of PPAR score. (A) The expression levels of PPAR‐related genes were displayed in the heatmap. (B) The distribution of PPAR score and clinical characteristics were shown. (C) GSEA analysis based on Hallmark gene set was used to explore the biological effect of PPAR score. (D, E) GSEA analysis based on GO gene set was used to explore the biological effect of PPAR score. (F, G) GSEA analysis based on KEGG gene set was used to explore the biological effect of PPAR score.

The expression level of 72 PPAR‐related genes were shown in Figure 2A. Then, the PPAR score was qualified based on the expression profile of 72 PPAR‐related genes using the ssGSEA algorithm (Figure 2B). GSEA analysis based on the Hallmark gene set indicated that the PPAR score might be involved in IL6/JAK/STAT3 signalling, bile acid metabolism, coagulation, fatty acid metabolism, myogenesis, complement, xenobiotic metabolism, allograft rejection, inflammatory response and oestrogen response late (Figure 2C). Meanwhile, GSEA analysis based on the GO gene set indicated that the activity of specific granule lumen, primary alcohol metabolic process and sterol homeostasis were positively correlated, while the activity of DNA packaging complex, nucleosome assembly and chromosome centromeric region were negatively correlated with PPAR score (Figure 2D, E); GSEA analysis based on the KEGG gene set indicated that the activity of complement and coagulation cascades, metabolism of xenobiotics by cytochrome P450 and drug metabolism cytochrome P450 were positively correlated, while the activity of cell cycle, oocyte meiosis and homologous recombination were negatively correlated with PPAR score (Figure 2F, G).

### 3.2 Construction of a prognostic model in the LUAD patients

**FIGURE 3:**
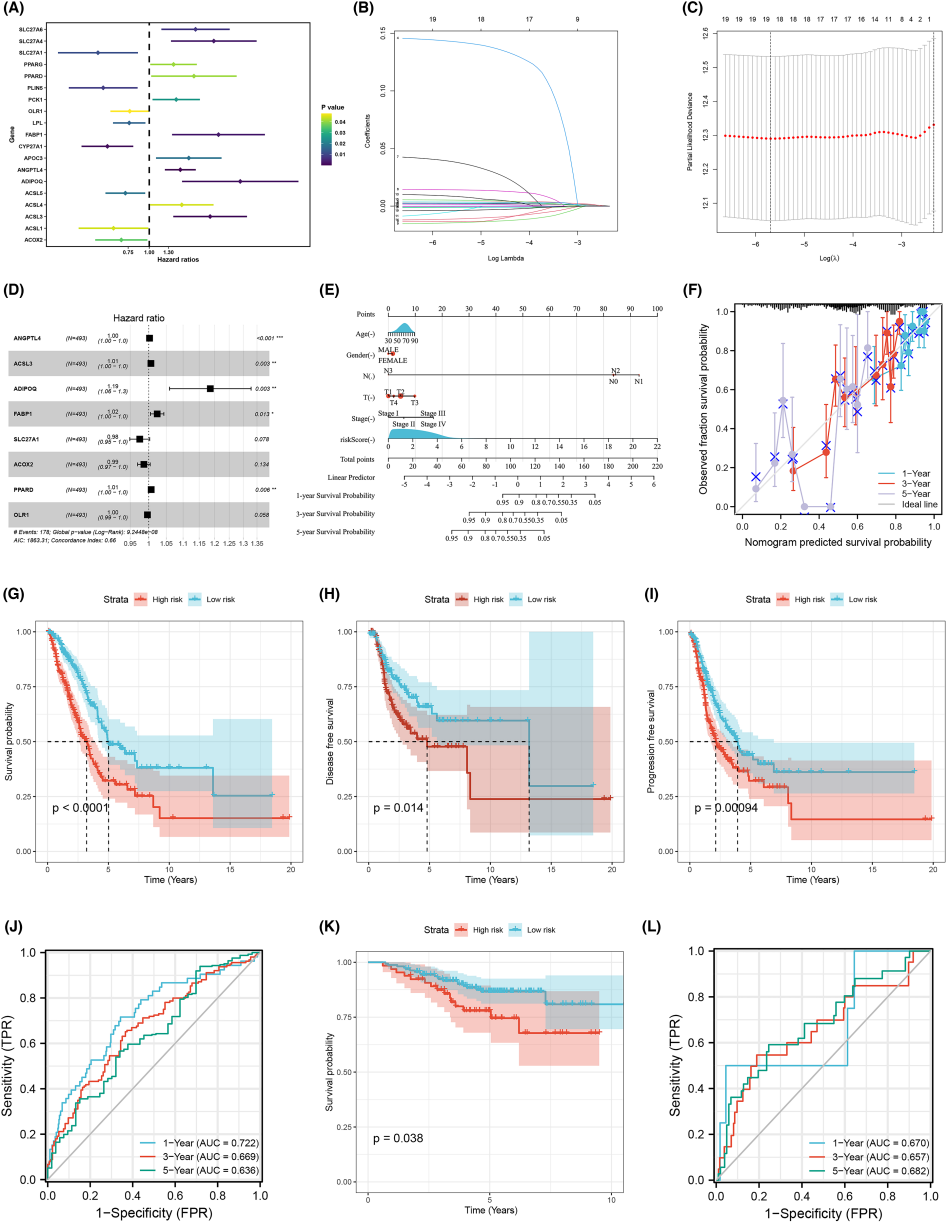
Construction of prognostic‐related PPAR‐gene signature. (A) Prognostic‐related PPAR genes were screened by Univariate Cox regression analysis. (B, C) LASSO regression analysis was used to prevent the overfitting effects of the model. (D) The screened genes were brought into multivariate Cox regression analysis and constructed the PPAR signature. (E) The nomogram was established to predict 1‐, 2‐, and 5‐year OS. (F) The calibration plot was used to be internal validation of the nomogram. (G–I) Kaplan–Meier curves of OS, DFS and PFS in the high‐ and low‐risk group. (J) The 1‐, 3‐ and 5‐year ROC curves of the risk score were shown (training cohort). (K) Kaplan–Meier curves of OS were shown (GSE31210). (L) The 1‐, 3‐ and 5‐year ROC curves of the risk score were shown (GSE31210).

Univariate Cox analysis was applied to identify PPAR‐related genes significantly correlated with patients survival, and these genes were included for further analysis (Figure 3A). Following this, an eight‐gene prognostic model was established based on the optimal value of λ through LASSO and multivariate Cox regression analysis (Figure 3B–D). The risk score was calculated with the formula of ‘Risk score = ANGPTL4 * 0.003 + ACSL3 * 0.006 + ADIPOQ * 0.171 + FABP1 * 0.023 + SLC27A1 * ‐0.025 + ACOX2 * ‐0.013 + PPARD * 0.007 + OLR1 * ‐0.004’. We devised a nomogram for predicting 1‐, 3‐ and 5‐year OS, incorporating factors such as the eight‐PPAR‐related gene signature, age, gender, N stage, T stage and clinical stage (Figure 3E). The calibration curves for 1‐, 3‐ and 5‐year OS suggest that the predicted probability of OS closely approximates the actual OS rates (Figure 3F). The prognostic value of the eight‐gene signature was also investigated by predicting OS, disease‐free survival (DFS) and progression‐free survival (PFS). The survival analysis indicated that the OS, DFS and PFS of LUAD patients in the low‐risk group were significantly longer than that in the high‐risk group (Figure 3G–I). The area under the curve (AUC) values of the signature for predicting 1‐, 3‐ and 5‐year survival in the TCGA‐LUAD cohort were 0.722, 0.699 and 0.636, respectively, whereas they were 0.670, 0.657 and 0.682 in the validation set (Figure 3J–L). Moreover, in the validation cohort, the OS duration for patients in the low‐risk group outlasted that of the high‐risk group.

### 3.3 Pathway enrichment analysis

**FIGURE 4:**
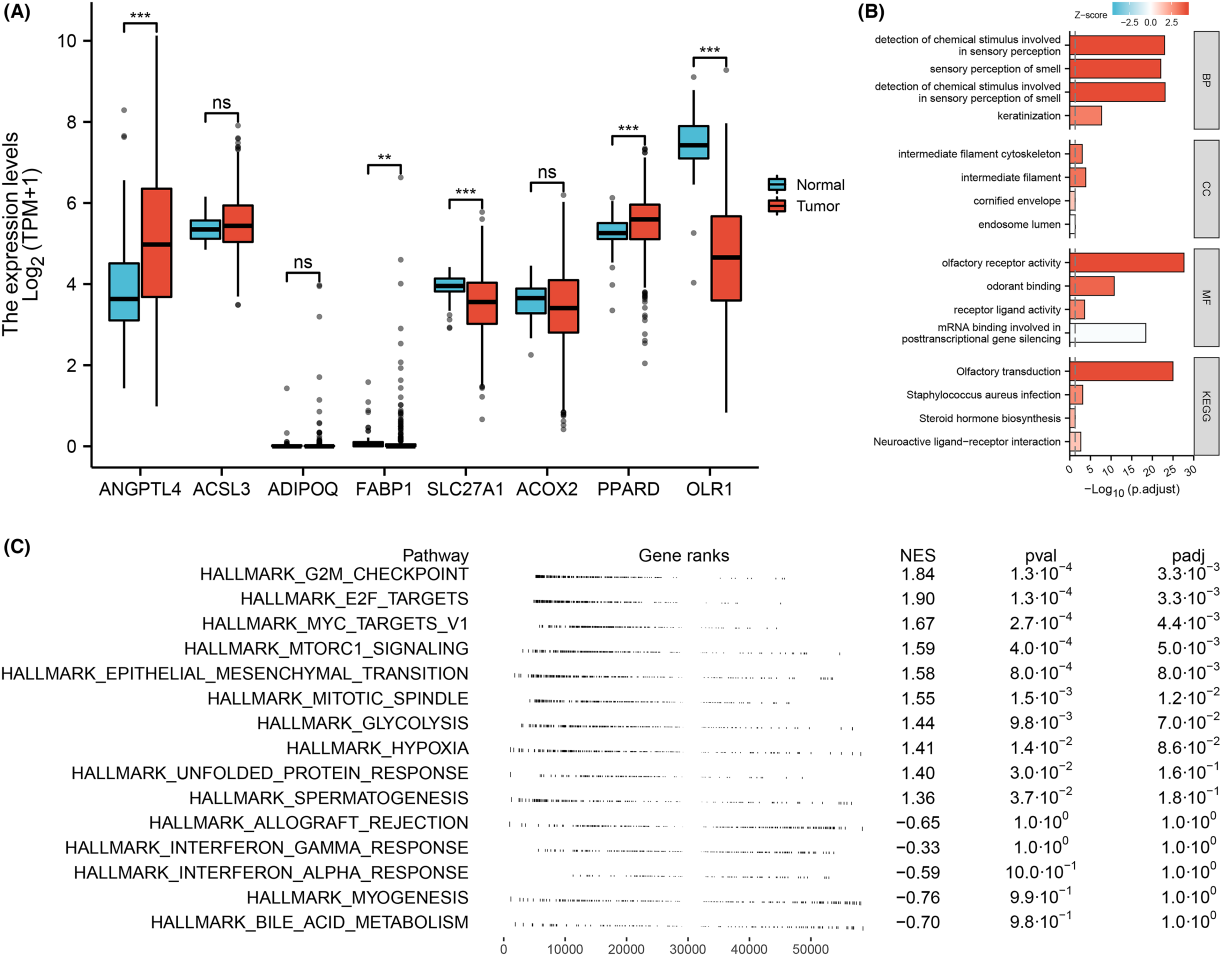
Gene enrichment analysis. (A) The expression levels of the selected genes between tumour and normal tissues, ns = *p* > 0.05, ** = *p* < 0.01, *** = *p* < 0.001. (B) The top 16 GO and KEGG signalling pathways in the high‐ and low‐risk groups. (C) The top 15 Hallmark signalling pathways in two groups.

Out of the eight genes incorporated in the model, the expression levels of five signature genes (ANGPTL4, FABP1, SLC27A1, PPARD, OLR1) exhibited significant differences between tumour and normal tissues. The expression levels of ANGPTL4 and PPARD were notably higher in tumour tissue, whereas the expression levels of FABP1, SLC27A1 and OLR1 were elevated in normal tissue (Figure 4A). The DEGs between high‐ and low‐risk groups were selected for enrichment analysis and some important pathways including the intermediate filament cytoskeleton, olfactory receptor activity and olfactory transduction pathway were enriched (Figure 4B). The top five most enriched pathways based on hallmark gene set in the high‐risk group were: G2M checkpoint, E2F targets, MYC targets V1, MTORC1 signalling and epithelial‐mesenchymal transition. The top five most enriched pathways in the low‐risk group were: interferon alpha response, interferon‐gamma response, allograft rejection, myogenesis and bile acid metabolism (Figure 4C).

### 3.4 Genomic mutation analysis

**FIGURE 5:**
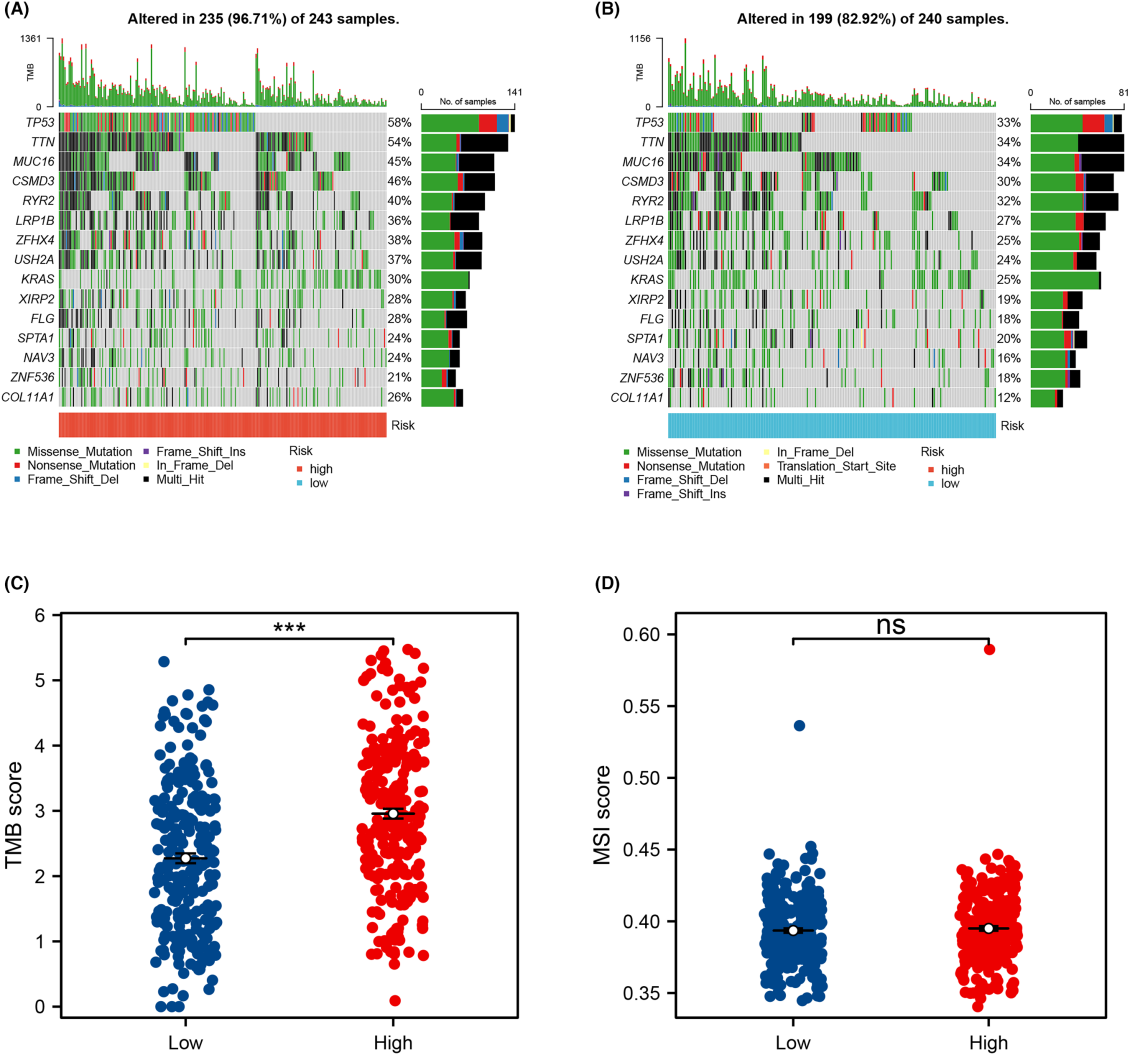
Genome instability analysis in LUAD. (A, B) The top 15 mutational genes were shown in the high‐ and low‐risk groups. (C) The TMB levels between high‐ and low‐risk groups, *** = *p* < 0.001. (D) The MSI levels between high‐ and low‐risk groups, ns = *p* > 0.05.

**FIGURE 6:**
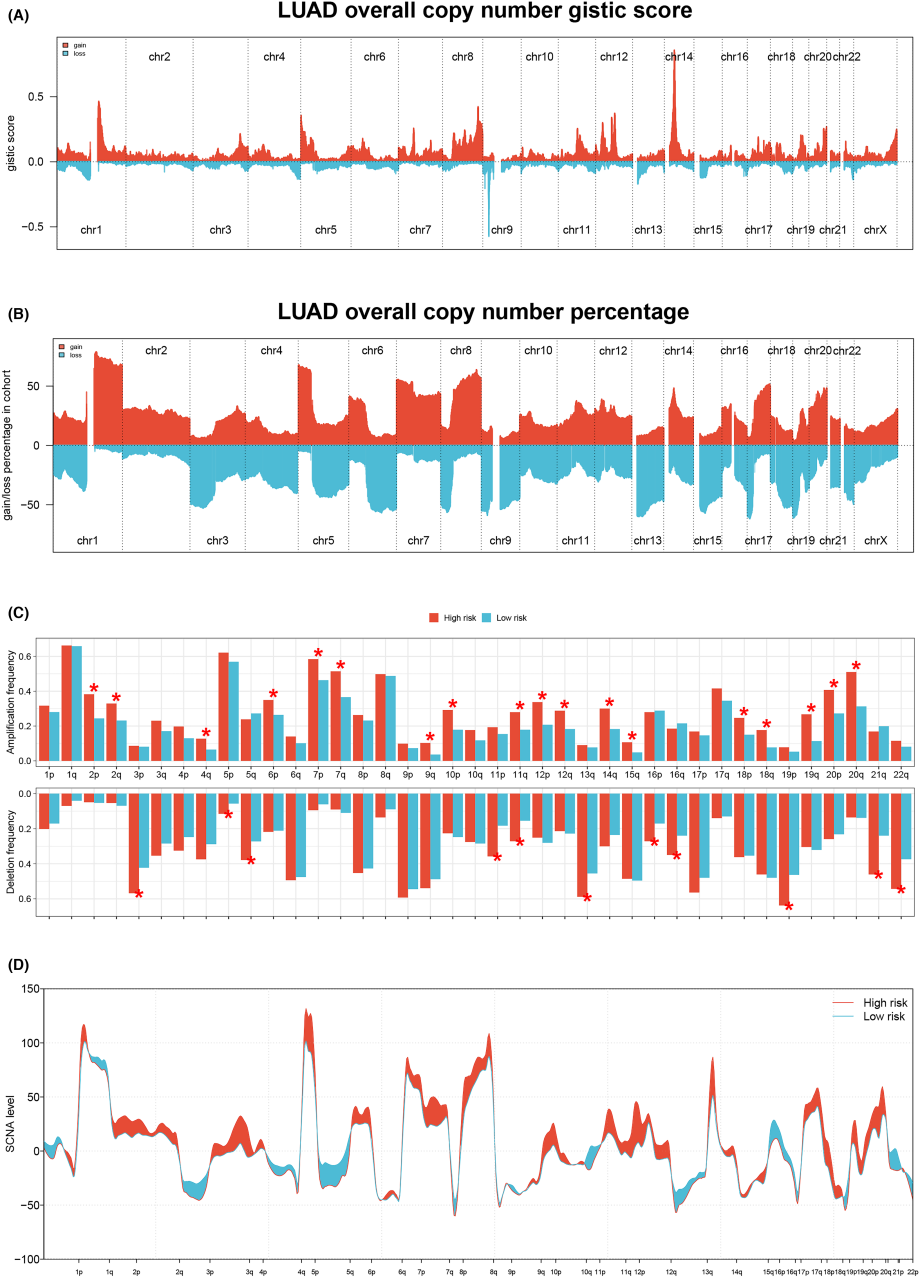
CNVs analysis in LUAD. (A, B) The distribution of copy number gistic score and percentage was shown. (C) The amplification and deletion frequency between the two groups were compared. (D) The line graph was used to display the SCNA levels in two groups.

The genomic mutation is an important role in cancer progression. We noticed that ZFHX4, LRP1B, RYR2, CSMD3, MUC16, TTN and TP53 had high mutation rates in both high‐ and low‐risk groups (Figure 5A, B). Moreover, LUAD patients classified in the high‐risk group exhibited higher levels of TMB compared to those in the low‐risk group, but not MSI (Figure 5C, D). Utilizing GISTIC 2.0, we identified amplified and deleted genomic regions in the LUAD cohort and presented the landscape of genomic characterizations (Figure 6A, B). Discrepancies were observed in the amplification and deletion of genomic regions, as well as the somatic copy number alteration (SCNA) levels, between the high‐ and low‐risk groups (Figure 6C, D). Patients in the high‐risk group displayed a tendency towards higher amplification frequencies in regions 2p, 2q, 4q, 6p, 7p, 7q, 9q, 10p, 11q, 12p, 12q, 14q, 15q, 18p, 18q, 19q, 20p and 20q. Furthermore, they demonstrated high deletion frequencies in regions 3p, 5p, 5q, 11p, 11q, 13q, 16p, 16q, 19p, 21q and 22q.

### 3.5 Immune microenvironment analysis

**FIGURE 7:**
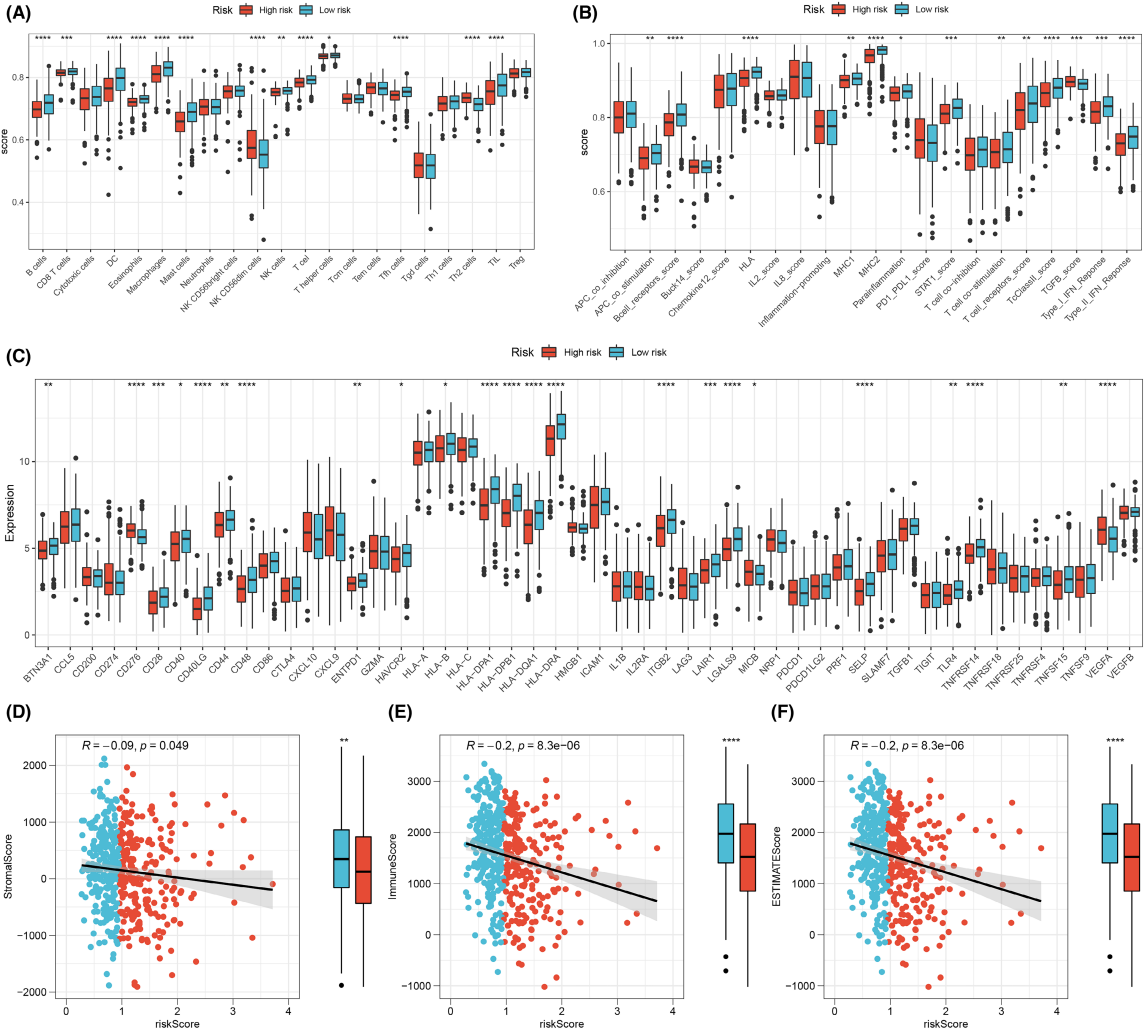
The immune infiltration levels in the LUAD microenvironment. (A, B) The score of immune cells and immune functions was compared between high‐ and low‐risk groups, * = *p* < 0.05, ** = *p* < 0.01, *** = *p* < 0.001, **** = *p* < 0.0001. (C) The expression level of immune checkpoints in two groups, * = *p* < 0.05, ** = *p* < 0.01, *** = *p* < 0.001, **** = *p* < 0.0001. (D–F) The ESTIMATE algorithm was used to calculate stromal, immune and estimate scores, ** = *p* < 0.01, **** = *p* < 0.0001.

The immune microenvironment frequently plays a pivotal role in tumour progression. Thus, we delved into the differences in the immune microenvironment between patients in high‐risk and low‐risk groups. Our findings revealed a diminished infiltration level of B cells, CD8 + T cells, dendritic cells, eosinophils, macrophages, mast cells, NK cells, T cells, helper T cells, follicular helper T cells and tumour‐infiltrating lymphocytes in the high‐risk group, while the levels of CD56dim NK cells and Th2 cells were elevated (Figure 7A). As for immune functionality, we noticed a decreased activity of antigen presenting cell (APC) co‐stimulation, B cell receptor scoring, Human Leukocyte Antigen (HLA), Major Histocompatibility Complexes (MHC) I, MHC II, parainflammation, STAT1 score, T cell co‐stimulation, T cell receptor score, T cytotoxic class II score, type I and II Interferon responses (Figure 7B). Furthermore, in comparison to the low‐risk group, the high‐risk group exhibited significantly lower levels of immune checkpoint family expression (Figure 7C). Additionally, a negative correlation was observed between the risk score and stromal score, immune score and estimate score (Figure 7D–F).

### 3.6 Immunotherapy and chemotherapy of PPAR‐signature

**FIGURE 8:**
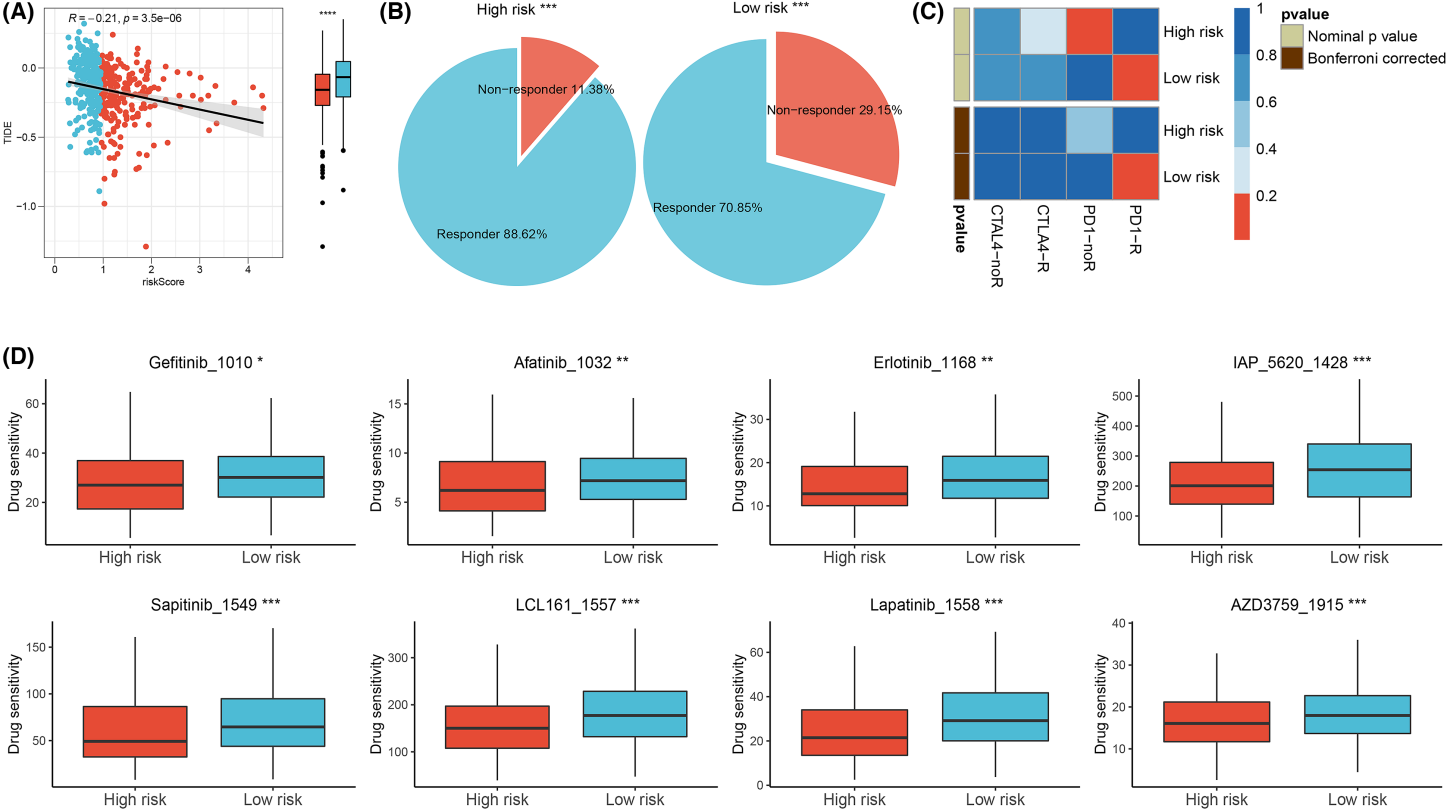
Immunotherapy and chemotherapy sensitivity analysis. (A, B) The TIDE scores and the number of patients who had responded to immunotherapy were compared between the two groups, *** = *p* < 0.001, **** = *p* < 0.0001. (C) SubMap analysis for PPAR‐signature in LUAD. (D) Box plots of estimated drug sensitivity for eight chemotherapeutic agents in the high‐ or low‐risk groups, * = *p* < 0.05, ** = *p* < 0.01, *** = *p* < 0.001.

According to the TIDE analysis, the TIDE level diminishes with increasing risk scores and more responders to immunotherapy were found in the high‐risk group (Figure 8A,B). Additionally, the Submap analysis suggested that patients in the low‐risk group may demonstrate a better response to anti‐PD‐1 therapy (Figure 8C). Moreover, we conducted an investigation on promising drugs (Gefitinib, Afatinib, Erlotinib, IAP_5620, Sapitinib, LCL161, Lapatinib, AZD3759), which are expected to be more beneficial for high‐risk patients (Figure 8D).

## 4 DISCUSSION

The population of LUAD exhibits a heterogeneous nature due to various genetic alterations including CNVs, single nucleotide variants, gene fusions and large‐scale chromosomal events.^24^ While drug therapies can target multiple cell lines, certain tumour subpopulations may display drug resistance, attributed to tumour heterogeneity. Tumour cells are capable of evading immune surveillance by manipulating the antigenic landscape, thus promoting their survival. Not only do they carry unique somatic mutation sequences, but these cells also serve as rich sources of neoantigens, deftly escaping robust antitumor immune responses.^25^ Tumour cells that overexpress PD‐L1 engage in negative feedback signalling to T cells, thus diminishing T cell cytotoxicity.^26^ However, through the application of antibodies that block negative feedback mechanisms—such as anti‐PD‐1, anti‐PD‐L1 and anti‐CTLA4—it's possible to induce tumour antigen‐specific immune responses and ultimately, eradicate the tumours.^27^ For LUAD patients resistant to these antibodies, targeting alternative immune checkpoints may offer a promising therapeutic approach.

PPAR signals play a critical role not only in lipid metabolism and glucose homeostasis but also in the modulation of immune response, cell proliferation, development, differentiation, apoptosis and motility.^28^ Precision oncology guided by genetic profiling, which targets specific genomic abnormalities, is at the forefront of cancer treatments. This approach takes into account aberrant gene mutations, abnormal cellular metabolism, rapid growth and low differentiation in cancerous cells.^29^, ^30^ In LUAD, PPAR signals are implicated in augmenting risk due to their connection to these dysregulated cellular processes and metabolic disorders.^31^ As evidence mounts that PPAR signals are altered during the process of carcinogenesis, the potential of targeting these pathways offers a promising avenue for both chemoprevention and therapeutic strategies.^32^

PPAR‐related genes have been identified to play crucial roles in various aspects of LUAD, including its development, growth, invasion, and metastasis. Our study identified eight key PPAR‐related genes ‐ ANGPTL4, ACSL3, ADIPOQ, FABP1, SLC27A1, ACOX2, PPARD and OLR1—and employed them to construct a prognostic signature for LUAD patients. Notably, ANGPTL4 has been significantly associated with various cancers,^33^, ^34^, ^35^ and ACSL3, crucial for fatty acid metabolism, exhibits altered expression levels in cancer, signalling aggressive tumour behaviour and a poor prognosis.^36^ The validity of this prognostic signature was thoroughly assessed using univariate, LASSO and multivariate Cox analyses, as well as ROC analysis. These evaluations confirmed that the prognostic signature, comprised of these eight PPAR‐related genes, can function as an independent prognostic factor for LUAD patients.

LUAD exhibits a highly heterogeneous immune microenvironment, with significant variance in the type and proportion of infiltrating immune cells.^37^ This heterogeneity is further evidenced by varying immune checkpoint expressions and differential immune cell and function scores. In our study, we examined the correlation between the PPAR signature and immune cell infiltration by comparing the immune cell scores of two risk groups. Our findings revealed significant differences in CD8 + T cells, B cells and NK cells between the high‐ and low‐risk groups. CD8 + T cells, vital constituents of lymphocyte infiltration in tumours, secrete various cytokines and have the ability to eliminate cancer cells.^38^ Activated B cells, another crucial component of the tumour microenvironment (TME), can enhance the effectiveness of immunotherapy, as demonstrated in breast cancer.^39^ Moreover, NK cells, known for their direct cancer cell killing capabilities, also play a key role in amplifying the immune response against tumours.^40^

In this study, we constructed a prognostic signature comprised of eight PPAR‐related genes, drawing from the TCGA‐LUAD cohort. The results indicated that a lower risk score in LUAD patients was associated with an extended survival period. Interestingly, we discovered that tumour‐specific gene expression levels were intrinsically tied to immune cell infiltration, thereby exerting a dual impact on the efficacy of immunotherapy. The insights gleaned from this research could potentially pave the way for breakthroughs in the treatment of LUAD.

However, our study still had several limitations. Firstly, we solely utilized public databases for signature validation, and did not analyse clinical samples, which would have offered more definitive evidence of its clinical applicability. Secondly, in order to substantiate our study's hypothesis and understand how the signature genes influence LUAD progression, it's necessary to conduct further in vivo and in vitro experiments. Thirdly, the integration of this signature with immunotherapy should be explored, to assess the magnitude of immunotherapy response and to discern the differential benefits between groups. Thus, to ensure broad clinical relevance, the prognostic PPAR signature warrants further investigation.

## AUTHOR CONTRIBUTIONS

**Wei Zhang:** Formal analysis (equal); investigation (equal); methodology (equal); visualization (equal). **Junhui Liu:** Conceptualization (equal); formal analysis (equal). **Xin Ren:** Data curation (equal); methodology (equal); writing – original draft (equal). **Zhengbin Zhang:** Formal analysis (equal); validation (equal). **Meilan Zhou:** Data curation (equal); software (equal). **Yuehua Li:** Conceptualization (equal); validation (equal); writing – review and editing (equal). **Jianjie Wang:** Investigation (equal); visualization (equal). **Quan Li:** Conceptualization (equal); software (equal). **Qi Zhu:** Formal analysis (equal); visualization (equal). **Gang Wu:** Project administration (equal); resources (equal).

## FUNDING INFORMATION

This work was funded by the Health Scientific Research Project of Health Commission of Hubei Province, China (WJ2023F054).

## CONFLICT OF INTEREST STATEMENT

None.

## Data Availability

All data can be obtained from the corresponding author based on reasonable requirements.
